# Effect of tillage and crop residue on soil temperature following planting for a Black soil in Northeast China

**DOI:** 10.1038/s41598-018-22822-8

**Published:** 2018-03-14

**Authors:** Yan Shen, Neil McLaughlin, Xiaoping Zhang, Minggang Xu, Aizhen Liang

**Affiliations:** 1 Institute of Agricultural Resources and Regional Planning, Chinese Academy of Agricultural Sciences, Beijing, China; 2 Ottawa Research and Development Centre, Agriculture and Agri-Food Canada, Ottawa, Ontario, Canada; 3 Northeast Institute of Geography and Agroecology, Chinese Academy of Sciences, Changchun, Jilin, China; 4 University of Chinese Academy of Sciences, Beijing, China

## Abstract

Crop residue return is imperative to maintain soil health and productivity but some farmers resist adopting conservation tillage systems with residue return fearing reduced soil temperature following planting and crop yield. Soil temperatures were measured at 10 cm depth for one month following planting from 2004 to 2007 in a field experiment in Northeast China. Tillage treatments included mouldboard plough (MP), no till (NT), and ridge till (RT) with maize (*Zea mays* L.) and soybean (*Glycine max* Merr.) crops. Tillage had significant effects on soil temperature in 10 of 15 weekly periods. Weekly average NT soil temperature was 0–1.5 °C lower than MP, but the difference was significant (*P* < 0.05) only in 2007 when residue was not returned in MP the previous autumn. RT showed no clear advantage over NT in increasing soil temperature. Higher residue coverage caused lower soil temperature; the effect was greater for maize than soybean residue. Residue type had significant effect on soil temperature in 9 of 15 weekly periods with 0–1.9 °C lower soil temperature under maize than soybean residue. Both tillage and residue had small but inconsistent effect on soil temperature following planting in Northeast China representative of a cool to temperate zone.

## Introduction

Conservation tillage systems can enhance sustainability of soil productivity by reducing soil erosion^1^ and increasing soil organic matter^2,3^. However, they have been shown to hinder soil warming in the early growing season in cold to temperate zones^4–6^.

Crop residue remaining on the soil surface in conservation tillage systems can decrease the rate of soil temperature change because surface residue both increases the reflection of incident solar radiation^7^, and acts as an insulating barrier between the soil surface and the warmer (or colder) atmospheric air above^8,9^. For example, the thermal conductivity of air-dry maize stalk mulch is about 20% that of the soil and the albedo is 0.18 compared to 0.08 for moist soil^10^.

Changes in soil thermal properties imparted by conservation tillage systems affect soil temperature^11^. Tillage can affect soil heat capacity and thermal conductivity and therefore thermal diffusivity (the ratio of the thermal conductivity to the heat capacity) by changing soil organic matter, bulk density, inter-aggregate contact and moisture content^12–14^. The soil thermal diffusivity was found to be 20–25% higher in the 5–15 cm layer^15^ and 37% higher in the 5–25 cm layer^16,17^ in no till compared to tilled soil. Therefore, more of the heat absorbed at the surface is transferred into deeper soil in the no till which leads to lower soil temperature in the near surface soil layers^18^.

Tillage can also affect soil temperature through changing soil surface micro topography. Radke^19^ reported that inclined ridge surfaces absorbed about 10% more solar radiation than flat surfaces. Ridges can drain more quickly and thus enhance drying of the seed zone, but they may also result in excessively wet soils in the ridge valleys.

Reduced maize emergence due to lower soil temperature of the seed zone was observed in conservation tillage systems compared to conventional tillage systems^20^. Similar trends were also reported on seed germination^21,22^, maximum leaf area index, crop growth rate^23^, and dry matter yield^24^. Lower yields with no till or reduced tillage systems compared to conventional tillage were reported by some researchers^25,26^ although others^27,28^ found similar or even higher yield in no till compared to conventional tillage. The contrasting beneficial aspects and the above mentioned negative side effects associated with conservation tillage systems sometimes present a dilemma for the farmers in cold to temperate regions.

Black soils in Northeast China are inherently productive and have been major maize- and soybean-producing areas since cultivated crops were introduced to the region about 100 years ago. Soil degradation has occurred because of intensive tillage leading to erosion and loss of organic matter^29^. Adoption of conservation tillage systems is imperative to maintain soil productivity. However, farmers in this region think conservation tillage, especially no till, results in lower soil temperature which they believe will significantly impede maize growth and decrease crop yields^30,31^. Scientific data on soil temperature under conservation tillage following planting are lacking in Northeast China, and it is difficult to convince farmers to adopt conservation tillage without these data. Therefore, it was deemed necessary to investigate the soil temperature regimes under different tillage systems in this region to quantify the effect of conservation tillage systems on soil temperature following planting. It is hypothesized that no till will decrease the soil temperature by increasing soil moisture and incident solar radiation reflection with more residue coverage.

The objectives of this study were to assess the difference in soil temperature following planting induced by three tillage systems, mouldboard plough (MP), no till (NT), and ridge till (RT), and two residue types, maize (*Zea mays* L.) and soybean (*Glycine max* Merr.), on a Black soil in Northeast China, and thus investigate whether conservation tillage with residue return leads to lower soil temperature following planting in a cool to temperate zone.

## Materials and Methods

### Site description

Soil temperature measurements were conducted on a tillage experiment established in autumn 2001 at Dehui city, Jilin province, Northeast China (44°12′N, 125°33′E, elevation 304 m). The soil studied was a typical Black Soil (Chinese soil classification) which was equivalent to Hepludolls in the USDA Soil Taxonomy System with 40% sand, 24% silt, and 36% clay content, 6.5 of pH, 15.7 g kg^−1^ of organic carbon and 1.3 g kg^−1^ of total Nitrogen^32^. Prior to establishing this tillage experiment, the land had been used for continuous maize production under conventional tillage management for more than 10 years. The mean annual temperature was 4.4 °C and the mean precipitation was 530 mm, with about 65% of the precipitation occurring from June through August.

### Experimental design

The tillage experiment was a split-plot randomized complete block design with four replications. The main plots were 10.4 m wide by 20 m long, and three tillage treatments, MP, NT, and RT were applied to the main plots. Each plot was split into two 5.2 m wide by 20 m long subplots that were planted with either maize or soybean in rotation and hence each crop occurred each year in each main plot. The maize-soybean rotation represented the current recommended farming practice in this region. All treatments were planted in north-south direction using a no till planter. Ridges for both MP and RT were constructed approximately 10 to 15 cm high during the first and the second cultivations (see Supplementary Table S1) in June.

### Residue Management

MP is the traditional tillage practice in the region, and farmers remove both maize and soybean residue after harvest and use it for fuel and animal feed. Excess residue not required for feed or fuel is disposed of by burning. We departed from this traditional practice, and left or replaced residue on the plots to replenish soil organic carbon. Maize was manually cut at 15 cm stubble height, cobs were manually removed and whole plant maize residue including stubble was left on the NT and RT plots after harvest. Maize residue was removed from the MP plots, the stubble was buried by mouldboard ploughing, and residue was manually replaced after autumn ploughing. Maize residue was not replaced in the MP plots in autumn 2006. During the late winter when the soil was still partially frozen, maize residue for all three treatments was cut into approximately 25 cm long pieces using heavily ballasted disk harrow. Mature soybean plants were removed from the plots, threshed by machine, and soybean residue was manually replaced on the corresponding plots; soybean residue was replaced on the MP plots after autumn ploughing.

### Soil temperature measurements

**Figure 1:**
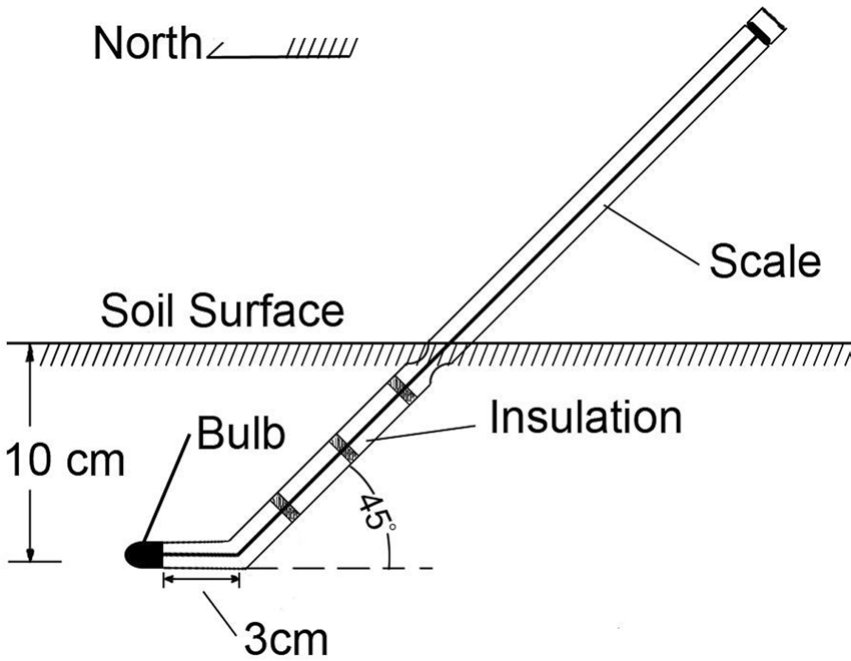
Side view of installation of bent stem earth thermometer. The thermometers were oriented in a north-south direction parallel to the maize and soybean rows. Direction north is to the left on the diagram as indicated by the arrow.

Soil temperature (2004–2007) was measured by bent stem earth thermometers (Wuqiang County Shengtong Instrument Factory, WQG-16) which were similar to regular liquid filled glass bulb thermometers except that the glass stem was bent 45° about 3 cm from the bulb (Fig. 1). Bent stem earth thermometer is a common instrument used to measure soil temperature in China^33^. The thermometers were graduated to 0.5 °C. The thermometers were placed in the same environment for a few days prior to the experiment to check consistency among the thermometer readings. Thermometers with readings departing more than 0.5 degrees from the mean were discarded. Maize and soybean were planted on May 06 and May 09 in 2004, April 28 and April 29 in 2005, and May 01 and May 02 in both 2006 and 2007, respectively. Soil temperature measurement started two days after soybean was planted and lasted for 32 days except in 2004 (21 days).

Bent stem earth thermometers were installed at in-row positions, north-south direction, with one thermometer in each plot. A small pit was dug with one wall of the pit left undisturbed. A rod was pushed into the undisturbed vertical soil wall of the pit at a depth of 10 cm from the surface to make a horizontal hole, and the thermometer was inserted into the hole. The rod had a slightly smaller diameter than that of thermometer probes to ensure that the thermometer probe made good contact with the undisturbed soil. After installation of the thermometers, the soil pits were backfilled and the displaced crop residue was manually returned to the soil surface. The depth of 10 cm was chosen because preliminary experiments showed that bent stem earth thermometers buried at shallower depth were unstable in the loose MP soil due to strong winds prevalent at the experimental site. The 10 cm depth was slightly below the seed depth of about 5–8 cm.

Soil temperatures were manually recorded at 9 am and 2 pm every day for approximately one month after planting except on days or half days when it rained and temperature data were not recorded (Table 1). A total of 42 to 62 (depending on the year) temperature measurements were collected from each plot each year for a grand total of 5016 temperature measurements for the four year experiment. 9 am and 2 pm were chosen according to the preliminary measurements, local experience, and labor availability. At 10 cm depth, the average daily soil temperature calculated from measurements at 9 am and 2 pm was 0.2 °C^34^, 0.6 °C^35^, and 0.6 °C^36^ lower than that calculated from measurements obtained with a data logger at 1-hour intervals over the day. This is within or similar to the precision of the thermometers adopted. Air temperatures with two replicates were measured by thermometers (Wuqiang County Shengtong Instrument Factory, WNG-01). These thermometers were located 1.5-m above the soil surface at the field site and installed in a three-sided shade to protect them from solar radiation.

**Table 1:** *P* values from repeated measures analysis of variance for testing effects of tillage, residue type and their interaction on soil temperature.

Year	Weekly period	Number of data (*n*)^†^	Tillage	Residue type	Tillage*residue type
2004	1	168	0.510	0.089	0.983
	2	168	0.021*	0.561	0.989
	3	168	0.022*	1.000	0.833
	na^a^	na	na	na	na
2005	1	168	0.257	0.003*	0.147
	2^b^	72	0.659	0.031*	0.225
	3^c^	144	0.500	0.002*	0.189
	4^d^	224	0.648	0.001*	0.191
2006	1	168	0.006*	0.145	0.069
	2	168	0.001*	0.003*	0.119
	3	168	0.001*	0.001*	0.308
	4^e^	264	0.000*	0.000*	0.054
2007	1	168	0.000*	0.001*	0.003*
	2	168	0.000*	0.006*	0.017*
	3 ^f^	120	0.000*	0.125	0.020*
	4 ^g^	216	0.000*	0.055	0.027*

^†^Soil temperatures measured at 9am and 2 pm each day were averaged as one datum. *The effect is significant (*P* < 0.05) according to repeated measures ANOVA.

^a^Na: not available; ^b^the period has 3 measuring days; ^c^the period has 6 measuring days; ^d^the period has 10 measuring days; ^e^the period has 11 measuring days; ^f^the period has 5 measuring days; ^g^the period has 9 measuring days.

### Residue coverage and soil moisture measurements

Residue coverage was measured on each plot prior to seeding using the line method^37^. Moisture of surface soil (0–15 cm) was determined by Time Domain Reflectometry (Model TRIME-FM, IMKO Manufacturing, Ettlingen, Germany) on selected dates before planting and during the soil temperature measurement periods. These measurements were required to investigate ancillary factors affecting soil temperature in addition to tillage practices.

### Statistical analysis

Daily soil temperatures were calculated by averaging the soil temperatures at 9 am and 2 pm. The daily temperatures were grouped into four one-week periods (Table 1) and weekly averages were calculated to reduce the day to day soil temperature fluctuations.

Repeated measures analysis of variance (ANOVA) was done for soil temperature in each one-week period and each of the four years using SPSS 16.0 (SPSS Inc., Chicago, IL, USA) with time as a within-subject factor and tillage and residue type as between-subjects factors^38^. Mauchly’s test of sphericity was done, and if *P* > 0.05, sphericity was met, and if not multivariate results or Greenhouse-Geisser correction results were used. Differences among treatment means (multiple comparisons) were determined using Tukey test and were considered significant at *P* < 0.05. Data were plotted using Origin 8.0 (OriginLab, Hampton, MA, USA).

### Data availability

All data generated or analysed during this study are included in this published article (and its Supplementary Information files).

## Results

### Daily soil temperatures

Soil temperature at 10 cm depth tracked the air temperature, but had lower diurnal amplitude (13.0–19.4 °C in 2004, Fig. 2) under all tillage systems compared to air temperature (13.0–27.5 °C in 2004, Fig. 2). There was considerable day to day variability in both soil and air temperature. The following results are for the one-week period soil temperature unless otherwise noted.

**Figure 2:**
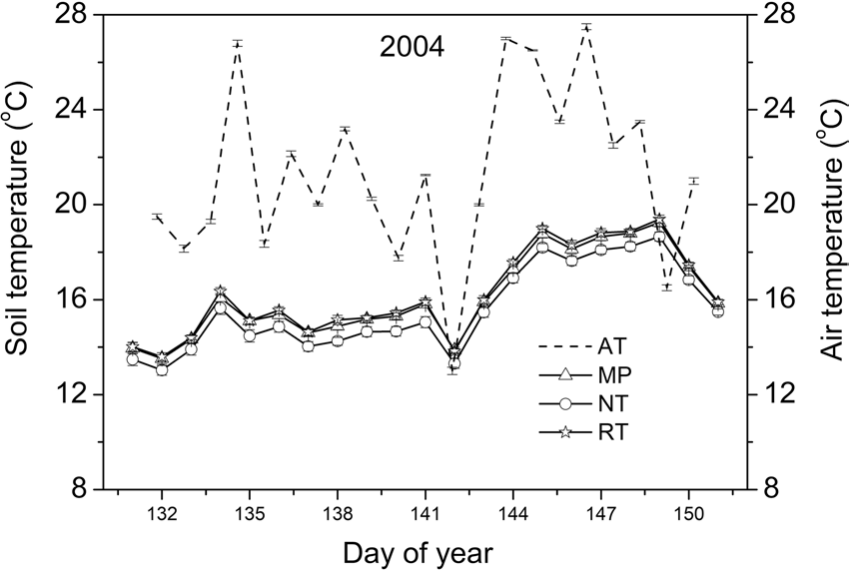
Daily soil and air temperature (mean ± standard error) in 2004. Data from maize and soybean plots for each tillage system were pooled. AT: air temperature; MP: mouldboard plough; NT: no till; RT: ridge till.

### Soil temperatures under different tillage systems

Repeated measures ANOVA showed that the effect of tillage on soil temperature was significant in 10 out of 15 weekly periods over the four years (*P* < 0.05), with the first week in 2004 and all four weeks in 2005 not significant (Table 1).

**Figure 3:**
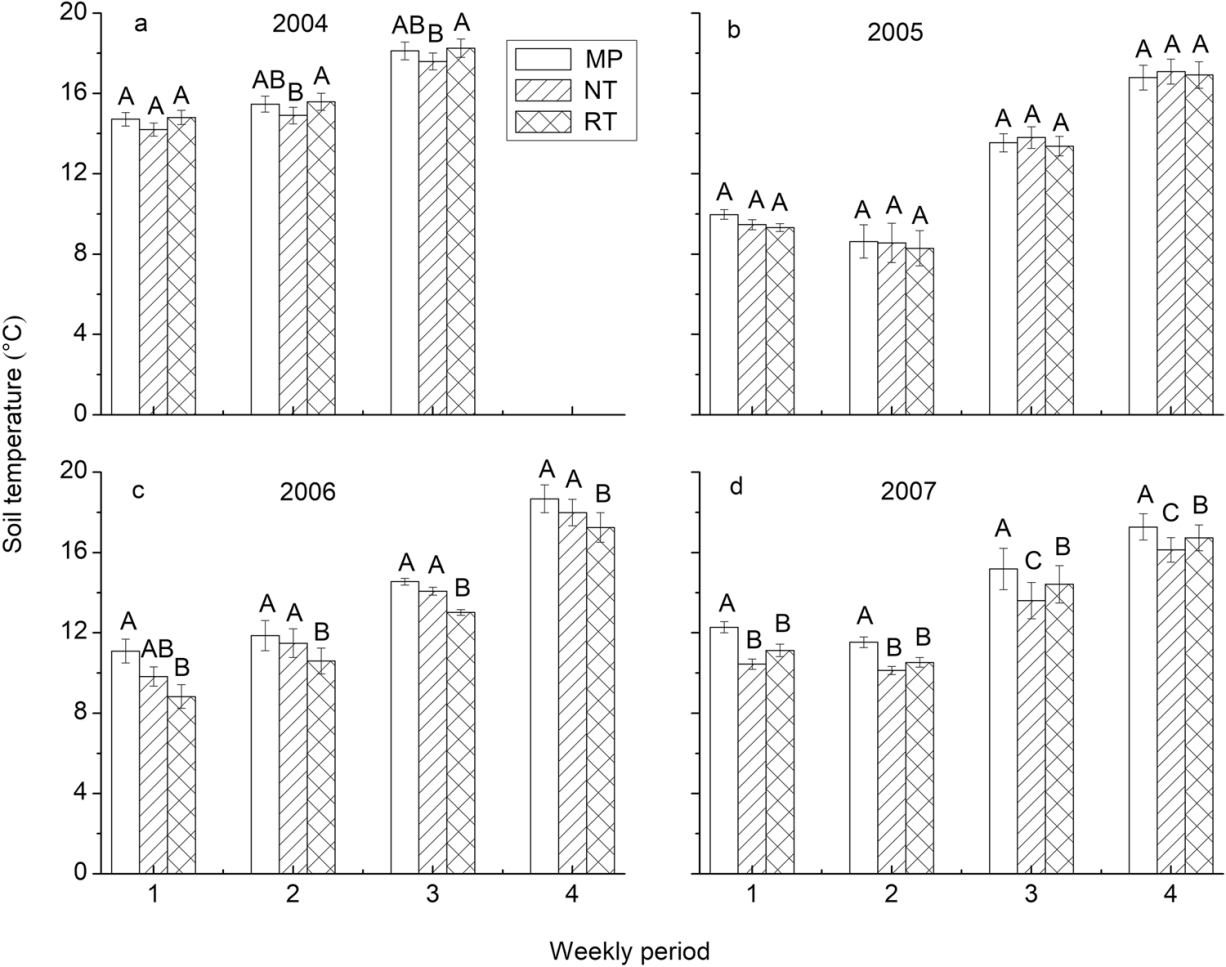
Weekly soil temperature (mean ± standard error) for different tillage systems in 2004 (**a**), 2005 (**b**), 2006 (**c**), and 2007 (**d**). Data from maize and soybean plots for each tillage system were pooled. Bars in the same period and capped by the same letter indicate the means are not significantly different (*P* > 0.05) according to Tukey test. MP: mouldboard plough; NT: no till; RT: ridge till.

The difference in soil temperature between tillage systems was not consistent over the four years (Fig. 3). In 2004 (Fig. 3a), there was significant effect of tillage on soil temperature in last two weekly periods (*P* < 0.05). In these two periods, soil temperature under NT was 0.6 °C lower than under MP and 0.7 °C lower than under RT. In the first period, there was a trend for slightly lower soil temperature under NT (14.2 °C) than under MP (14.7 °C), and slightly higher soil temperature under RT (14.8 °C) than under MP (14.7 °C). In 2005 (Fig. 3b), soil temperature showed the trend of MP > NT > RT in the first two weekly periods but NT > RT > MP in the last two weekly periods, but with no significant difference among three tillage systems (*P* > 0.05). Soil temperature under NT was significantly lower than under MP in the first weekly period of 2006 and all four weekly periods in 2007 (*P* < 0.05), with the average difference of 0.6 °C in 2006 and 1.5 °C in 2007 (Fig. 3c,d). Soil temperature under RT was significantly lower than MP in all four periods both in 2006 and 2007 (*P* < 0.05), with the average difference of 1.5 °C in 2006 and 1.0 °C in 2007 (Fig. 3c,d).

### Soil temperatures under different residue types (maize and soybean)

The effect of residue type was significant in 9 out of 15 periods over the four years (Table 1). Similar to tillage effect, the effect of residue type (maize or soybean) on soil temperature was not consistent over the four years.

**Figure 4:**
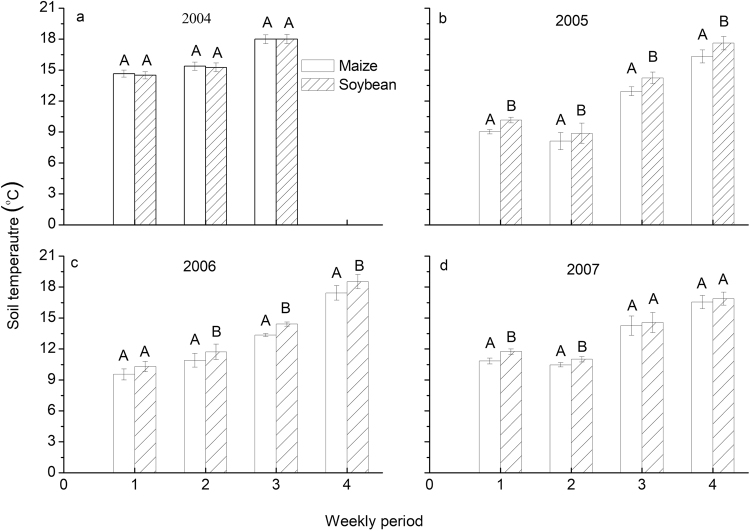
Weekly soil temperature (mean ± standard error) for maize residue and soybean residue in 2004 (**a**), 2005 (**b**), 2006 (**c**), and 2007 (**d**). Data from three tillage systems for each residue type were pooled. Bars in the same period and capped by the same letter indicate the means are not significantly different (*P* > 0.05) according to Tukey test.

Residue type had no significant effect on soil temperature in 2004. Soil temperature for maize residue was almost the same as that for soybean residue (Fig. 4a). Residue type showed significant effect on soil temperature for all four periods in both 2005 and 2006, with the mean difference of 1.1 °C in 2005 and 1.0 °C in 2006 (Fig. 4b,c), respectively. In 2007, effect of residue type on soil temperature was significant only for the first two periods, with the mean weekly-period difference of 0.5 °C (Fig. 4d).

## Discussion

### Effect of tillage system on soil temperature

In this study, the difference in soil temperature among the three tillage systems varied over the four years. Soil temperature under NT was lower than under MP in 13 of the 15 weekly periods over the four years, with four of these weekly periods showing significant difference (*P* < 0.05). This may be in part due to the significantly higher residue coverage under NT than under MP. Residue coverage (mean of maize and soybean) in NT plots was higher than in MP plots by 124% in 2004, 78% in 2005, and 161% in 2006 (Table 2). Maize residue was not replaced for MP in autumn 2006 to comply with local custom, and the lack of insulation by residue likely contributed to the much higher (1.5 °C) soil temperature under MP than under NT in the following spring in 2007.

**Table 2:** Percent of soil surface covered by crop residue under the three tillage systems (*n* = 4).

Residue/Tillage	Residue coverage (%)			
	2004	2005	2006	2007
**Maize**				
MP	20.7 ± 4.2b	51.2 ± 23.0b	39.2 ± 25.7b	0^†^
NT	53.0 ± 7.9a	91.0 ± 11.7a	86.0 ± 3.4a	89.0 ± 3.9a
RT	23.5 ± 5.4b	83.2 ± 10.2a	85.7 ± 10.2a	81.0 ± 6.4b
**Soybean**				
MP	33.5 ± 11.5B	30.0 ± 25.3A	19.7 ± 3.9B	19.2 ± 3.3C
NT	68.7 ± 9.0A	53.2 ± 10.4A	68.0 ± 24.4A	71.7 ± 5.4A
RT	48.2 ± 15.6AB	56.5 ± 12.3A	68.5 ± 6.0A	59.2 ± 11.6B

MP: mouldboard plough; NT: no till; RT: ridge till

Numbers are means of percent residue coverage ± Standard Deviation;

Means in the same column and residue, and followed by the same letter are not significantly different (*P* > 0.05) according to the Duncan test;

^†^Maize residue was not returned after autumn plough in 2006.

Residue on the surface can reflect solar radiation and insulate the surface soil from the atmosphere, and thus decrease the warming rate of soil^10^. The linear negative relationships between soil temperature and residue coverage (Table 3) supports this hypothesis. For both maize and soybean, residue coverage showed a significantly negative relationship with soil temperature except in 2005, with 0.0112 to 0.0293 °C decrease in soil temperature for a percent increase of residue coverage (Table 3). High surface residue coverage of 50.2% and 30.0% for maize and soybean respectively was found under MP in 2005 (Table 2). This high residue coverage likely lowered the soil temperature under MP, and thus reduced the difference in soil temperature between MP and the other two tillage systems (Fig. 3). Soil moisture was measured at different times throughout the growing seasons of 2004 to 2007, but unfortunately, most of these measurement times did not coincide with the temperature measurement period. Soil moisture under NT was significantly higher than under MP in 9 out of 11 measured times during 2004–2007 (Table 4). We can speculate from this result to some extent that soil moisture under NT was higher than under MP during the soil temperature-measuring period. Soil temperature was negatively correlated with soil moisture content^39^ because wetter soil requires more absorbed solar energy to warm the soil due to the higher specific heat capacity^40^, higher thermal conductivity^41–43^, and energy required to evaporate excess soil water^44^.

**Table 3:** Coefficients for slopes of linear regression equations relating mean soil temperature and residue coverage (*n* = 12).

Year	Residue type	
	Maize	Soybean
2004	−0.0230 (0.0007)*	−0.0177 (0.0074)*
2005	−0.0101 (0.0103)	0.0208 (0.0109)
2006	−0.0293 (0.0075)*	−0.0057 (0.0081)
2007	−0.0220 (0.0036)*	−0.0112 (0.0044)*

Soil temperature in this analysis refers to the average soil temperature over the measurement period in each year of each plot.

Units of the slopes are degrees C per percent residue coverage

Slopes are followed by the standard error of the estimate

Slopes followed by an asterisk indicate that they are significant (*P* < 0.05).

**Table 4:** Soil moisture (%) on selected dates under different tillage systems.

Date	MP	NT	RT
2004-4-14	19.7 ± 2.9a	22.2 ± 2.2a	20.3 ± 2.2a
2004-4-16	18.5 ± 1.4b	20.9 ± 2.2a	20.7 ± 1.7a
2005-4-27	24.4 ± 1.3b	29.0 ± 1.7a	23.4 ± 1.2b
2006-4-26	21.3 ± 2.5c	28.5 ± 2.3a	24.3 ± 3.3b
2006-4-27	19.8 ± 2.4c	27.8 ± 2.8a	23.0 ± 2.4b
2006-4-30	22.8 ± 2.9c	30.4 ± 2.6a	26.8 ± 2.3b
2007-4-29	18.1 ± 1.4c	28.1 ± 1.5a	24.4 ± 4.3b
2007-4-30	22.8 ± 1.6c	28.1 ± 1.7a	25.5 ± 2.6b
2007-5-21	24.1 ± 1.0b	25.5 ± 1.3a	24.9 ± 1.0ab
2007-5-31	22.5 ± 2.6b	25.0 ± 2.1a	26.1 ± 1.6a
2007-6-26	15.4 ± 4.0a	18.2 ± 3.7a	18.9 ± 3.8a

MP: mouldboard plough; NT: no till; RT: ridge till

Numbers are means of average soil moisture ± Standard Deviation;

Means in the same row and followed by the same letter are not significantly different (*P* > 0.05) according to the Duncan test.

Soil temperature under RT was significantly higher than NT in 4 weekly periods (last two weekly periods both in 2004 and 2007) but significantly lower than NT in 2 weekly periods (the second and third weekly period in 2006) (*P* < 0.05), while the difference was not significant in the other 9 weekly periods (*P* > 0.05, Fig. 3). The residue coverage under RT was significantly lower than under NT in 2004 and in 2007 (Table 2), which likely contributed to higher soil temperature under RT than under NT in these two years (Fig. 3a,d). Ridges under RT formed in the previous year were about 5 cm high at planting due to the flattening by wind and rainfall, and this dimension did not change much over the four years. Ridges can increase radiation exposure in the seed zone^19^ and allow water to quickly drain off the ridge and thus increase soil-drying rate in the seed zone^15^. This may also contribute to higher soil temperature under RT than under NT. The residue coverage under RT and NT was similar both in 2005 and 2006 (Table 2). Soil moisture under NT was significantly higher than under RT prior to planting in 2005 and 2006 (Table 4), however, soil temperature under NT was 0.2 °C higher in 2005 and 0.9 °C higher in 2006 than under RT (Fig. 3b,c). Soil moisture affects both specific heat capacity and thermal conductivity of the soil^40,42^. Thus, tillage affects soil temperature both by influencing residue amount left on the soil surface, and by affecting soil thermal parameters. The effect of tillage system on soil thermal properties needs further study to provide a more complete understanding.

Soil temperature under RT was significantly lower than MP in all four weekly periods both in 2006 and 2007 (*P* < 0.05), but with no significant difference in 2004 and 2005 (Fig. 3). This result is consistent with the results of residue coverage and soil moisture. Residue coverage under RT was significantly higher than that under MP in 2006 and 2007 (*P* < 0.05) with much less difference in 2004 and 2005 (Table 2). Meanwhile, soil moisture under RT was significantly higher than RT in six out of eight measured times in 2006–2007 but only significant in one out three measured times in 2004–2005 (*P* < 0.05; Table 4). RT was considered as an attractive alternate conservation tillage system in northern regions of the United States, because of higher soil temperature than under NT^45^. Similar soil temperatures within the seed zone under RT and MP were reported by Stone *et al*.^46^, which was a little different from the results in this study. This disagreement may be caused by crop residue difference between the two studies. In Stone *et al*.^46^, RT consisted of two kinds of residue management, one with and one without autumn chopping of maize stalks. In our study, residue coverage under RT and NT was similar but much higher than the 30% required for conservation tillage in western countries^47^. Therefore, the insulating effect of residue enlarged the difference in soil temperature between RT and MP and weakened the advantage of RT over NT. In addition, soil temperature in Stone *et al*.^46^ was measured at 5-cm soil depth, which should have a greater sensitivity to periodic changes in air temperature and surface soil temperature than soil temperature measured at 10-cm soil depth in our study^48^.

### Effect of residue type (maize and soybean) on soil temperature

Soil temperature for maize residue was consistently lower than for soybean residue (Fig. 4). This may be caused by several reasons. Firstly, maize residue coverage was higher than soybean except in 2004 (Table 2), and thus provided more insulation and impeded absorption of solar energy and heat transfer from the air. This is consistent with the slopes of the regression equations in Table 3, where slopes for soybean residue were much smaller than for maize residue. Secondly, the line method of measuring residue coverage indicates the presence or absence of a residue fragment at each measurement point^46^, but does not provide information on the size or thickness of the fragment, both of which affect the insulating properties^48^. Maize residue is generally much larger and thicker than soybean residue and the insulating properties of maize and soybean residue at the same coverage as indicated by the line method are likely quite different. In addition, maize residue decomposes much slower than soybean residue^49^, with much of the maize residue remaining at the end of the measurement period resulting in a prolonged insulating effect.

In 2004, soil temperature for maize residue was slightly higher than for soybean residue (Fig. 4a). In this year, higher soybean residue coverage than maize residue was found in all three tillage systems (Table 2), which supports the above arguments that the amount of residue played an important role in influencing soil temperature.

### Interaction effect of tillage and residue type

In this study, it is difficult to determine whether tillage practice or residue type played a more important role in determining soil temperature. Repeated measures ANOVA showed that tillage and residue type significantly affected soil temperature in 10 and 9 out of 15 weekly periods, respectively (Table 1), but their effects were significant in different weekly periods over the four years. Although the interaction effect of tillage and residue type on soil temperature was not significant except in 2007 (Table 1), both tillage and residue type affect residue coverage, which in turn has a strong effect on soil temperature. Therefore, the difference caused by residue type may mask the effect of tillage, resulting in no significant difference in soil temperature between tillage systems. In our experiment, residue was manually replaced after mouldboard ploughing to protect the soil and improve soil organic carbon for MP. This was a departure from traditional mouldboard ploughing treatment, and may be a major reason why our results did not show as large difference in soil temperature among tillage systems as some other studies.

### Long-term effect that tillage systems may exert on soil temperature

Soil thermal properties can be changed by conservation tillage systems, and thus affect soil temperature^11^. The small difference in soil temperature among MP, RT and NT (Fig. 4) could be caused by many tillage-induced factors, such as soil organic matter, bulk density, inter-aggregate contact and moisture content^12–14^. Effects of tillage on some of these factors have been reported in previous studies on the same field experiment and are summarized as follows. There were no significant differences (*P* > 0.05) in SOC storage on equivalent soil mass basis among tillage treatments after three years of tillage experiment^32^. After five years, the SOC in >1000 μm aggregate at 0–5 cm depth was significantly higher in NT soil (1.32 g C kg^−1^) than in RT (0.6 g C kg^−1^) and MP soil (0.25 g C kg^−1^)^50^. Furthermore, tillage treatments, after six years, influenced soil porosity significantly. Both pores with diameter <30 μm and 30–100 μm under NT were smaller than those under MP in the soil profile, but with little difference in proportion of large pores (>100 μm) between NT and MP^51^. Dye tracer and double-ring infiltrometer techniques showed that NT had better development of pore structure and more biological pores, and presented better preferential flow character after six years^52^. In addition, NT increased the soil penetration resistance, especially at the soil depth of 2.5–17.5 cm; NT increased the bulk density of 5–20 cm soil layer significantly after eight years^53^. From these results, it is evident that long-term NT gradually affects soil physical and chemical properties thus affecting soil thermal properties. However, it likely takes time to reach changes in soil conditions sufficient to cause the variation of soil temperature.

## Conclusion

This study provided insights into changes of soil temperature following planting induced by tillage practices and residue types in Northeast China representative of cold to temperate zones. Soil temperature was affected by both tillage and residue type, however, the effects of both tillage and residue type on soil temperature were not consistent over the four years. Soil temperatures under NT were 0.5–0.9 °C lower than under MP during the four periods of 2004–2007, but with significant difference only in 4 out of 15 weekly measurement periods. All four of these periods were in 2007 where residue for MP was not replaced the previous autumn. Mean soil temperature under RT was 0.2 °C and 0.9 °C lower than under NT in 2005 and 2006 respectively, and 0.7 °C and 0.6 °C higher than under NT in 2004 and 2007 respectively, indicating that RT in this study did not show any obvious advantage in increasing soil temperature over the NT system. Soil temperature in plots with maize residue was lower than in plots with soybean residue. Residue coverage had a strong effect on soil temperature; significant negative linear relationship (*P* < 0.05) was found between soil temperature and maize residue coverage in three of the four years, while for soybean residue coverage, the relationship was variable and much weaker over the four years. Reducing residue return by harvesting a portion of the residue for other purposes such as bioenergy while returning enough residue to protect the soil from erosion and maintain soil health may promote higher soil temperatures in a conservation tillage system.

## Electronic supplementary material

supplementary information
