# Supplementary material for: Effect of tillage and crop residue on soil temperature following planting for a Black soil in Northeast China
Source: Sci Rep. 2018 Mar 14;8:4500. doi: 10.1038/s41598-018-22822-8 (PMC5852035; doi:10.1038/s41598-018-22822-8)
Supplement: Supplementary file 1 — supplementary information [file 41598_2018_22822_MOESM1_ESM.pdf]

Effect of tillage and crop residue on soil temperature following planting for a Black soil in Northeast China

Yan Shen[1], Neil McLaughlin[2], Xiaoping Zhang[3], Minggang Xu[1], & Aizhen Liang[3,4*]

[1] Institute of Agricultural Resources and Regional Planning, Chinese Academy of Agricultural Sciences, Beijing, China

[2] Ottawa Research and Development Centre, Agriculture and Agri-Food Canada, Ottawa, Ontario, Canada

[3] Northeast Institute of Geography and Agroecology, Chinese Academy of Sciences, Changchun, Jilin, China

[4] University of Chinese Academy of Sciences, Beijing, China

Supplementary Information

Table S1. Field management operations and their timing under three tillage treatments each year.

Field operation[†]	MP	NT	RT
Shallow cultivation	Late April	-	-
Sowing and fertilization	Early May	Early May	Early May
Herbicide	Early May	Early May	Early May
First cultivation	Late May	-	Late May
Second herbicide if necessary	Late May	Late May	Late May
Hand hoeing and thinning	Mid June	Mid June	Mid June
Second cultivation and ridge building	Mid to late June	-	Mid to late June
Compound fertilizer side-dress	Mid to late June	-	Mid to late June
Final cultivation and ridge building	Late June	-	Late June
Compound fertilizer	-	Early to mid July	-
Harvest	Early October	Early October	Early October
Aboveground residue returning	-	Early October	Early October
Fall plough and cultivation	Late October	-	-
Aboveground residue returning	Late October	-	-

MP: mouldboard plough; NT: no till; RT: ridge till

†: operations for maize and soybean were similar but there were a few days difference between the dates of each operation
